# A systematic review of randomized control trials of HPV self-collection studies among women in sub-Saharan Africa using the RE-AIM framework

**DOI:** 10.1186/s43058-021-00243-5

**Published:** 2021-12-15

**Authors:** Ucheoma Nwaozuru, Chisom Obiezu-Umeh, Chisom Obi-Jeff, Thembekile Shato, Titilola Gbaja-Biamila, David Oladele, Ifeoma Idigbe, Joseph Tucker, Oliver Ezechi, Juliet Iwelunmor

**Affiliations:** 1 College for Public Health and Social Justice, Saint Louis University, 3545 Lafayette, Ave, Saint Louis, Missouri 63104 USA; 2 Direct Consulting and Logistics, Federal Capital Territory, Abuja, Nigeria; 3 Implementation Science Center for Cancer Control and Prevention Research Center, Brown School, Washington University in St. Louis, One Brookings Drive, Campus Box, 1196, St. Louis, Missouri 63130 USA; 4 Clinical Sciences Division, Nigerian Institute of Medical Research, Medical Compound, 6 Edmund Crescent, Yaba, Lagos, Nigeria; 5 University of North Carolina at Chapel Hill, Chapel Hill, NC 27599 USA; 6 London School of Hygiene and Tropical Medicine, London, UK

**Keywords:** HPV testing, Self-collection, Cervical cancer screening, Sub-Saharan Africa, Implementation science, Women, RE-AIM

## Abstract

**Introduction:**

Self-collection of samples for HPV testing may increase women’s access to cervical cancer screening in low- and middle-income settings. However, implementation remains poor in many regions. The purpose of this systematic review was to examine implementation data from randomized controlled trials evaluating human papillomavirus (HPV) self-collection testing among women in sub-Saharan Africa using the RE-AIM (Reach, Efficacy/Effectiveness, Adoption, Implementation, and Maintenance) framework.

**Methods:**

We searched four electronic databases (PubMed, CINAHL, Web of Science, and Global Health) for pragmatic randomized controlled trials that promote HPV self-collection among women in sub-Saharan Africa. Study selection and data extraction were conducted according to the PRISMA (Preferred Reporting Items for Systematic Reviews and Meta-analyses) checklist. Two researchers independently extracted information from each article using a RE-AIM data extraction tool. The reporting of RE-AIM dimensions was summarized and synthesized across included interventions.

**Results:**

We identified 2008 citations, and eight studies were included. These reported on five unique interventions. The five interventions were conducted in five countries: Cameroon, Ethiopia, Kenya, Nigeria, and Uganda. Intervention reach (80%) was the most commonly reported RE-AIM dimension, followed by adoption (56%), efficacy/effectiveness (52%), implementation (47%), and maintenance (0%). All the interventions described increased uptake of HPV testing among study participants (effectiveness). However, the majority of the studies focused on reporting internal validity indicators such as inclusion criteria (100%) and exclusion criteria (100%), and few reported on external validity indicators such as participation rate (40%), intervention cost (40%), staff selection (20%), and cost of maintenance (0%).

**Conclusions:**

Our review highlights the under-reporting of external validity indicators such as participation rate, intervention, and maintenance costs in studies of self-collection for HPV testing among women in SSA. Future research should focus on including factors that highlight internal validity factors and external validity factors to develop a greater understanding of ways to increase not only reach but also implementation and long-term maintenance of these interventions. Such data may advance the translation of HPV interventions into practice and reduce health disparities in SSA. Findings highlight the need for innovative tools such as participatory learning approaches or open challenges to expand knowledge and assessment of external validity indicators to ultimately increase the uptake of HPV testing among women in SSA.

**Supplementary Information:**

The online version contains supplementary material available at 10.1186/s43058-021-00243-5.

Contributions to the literature

- With poor screening for cervical cancer among women in sub-Saharan Africa, HPV self-collection provides a convenient and innovative tool to promote testing for HPV, the major cause of cervical cancer among women. Implementation evidence to guide the translation and dissemination of effective approaches is needed.
- We examined the external and internal validity of HPV self-collection interventions using the RE-AIM framework, which are critical factors to inform intervention translation and implementation.
- Our review found limited reported data on external validity indicators, which are important to determine generalizability and bridge the research to practice gap.
- Future research should focus on balancing reporting of external and internal validity indications and should be designed to highlight the translatability of empirical evidence to real-world settings.

## Background

Human papillomavirus (HPV) causes cervical cancer and can be prevented by screening [1]. However, the implementation of cervical cancer screening programs has been difficult in sub-Saharan Africa (SSA), where one third of deaths due to cervical cancer occur [2]. Individual (i.e., lack of knowledge and awareness about cervical cancer and preventive service, low-risk perception) [3–8], social (i.e., the stigma associated with acquiring disease via sexual intercourse, partner disapproval, cultural and religious beliefs) [7, 9], and structural factors (i.e., high service cost, distance to the health facility, poor and limited training of health providers to conduct test) [1, 10, 11] limits effective pap smear screening among women in the region. However, HPV testing provides a new method for cervical cancer screening.

HPV testing and treatment have been shown to reduce cervical cancer incidence and mortality in low-resource settings [12]. HPV testing has excellent test characteristics, a longer screening interval of 5 years, and for women aged 30 years and older as recommended by the World Health Organization (WHO) [13–15]. There are two approaches to HPV testing. The physician-provided approach entails the collection of the vaginal samples by the healthcare provider which are then sent to the laboratory for testing. HPV testing also allows for self-collection of vaginal samples [16], a method where women collect samples themselves and send them to the clinic or laboratory for testing. HPV self-collection may decrease stigma, lack of privacy, and inconvenience and improve access in remote areas [17–19]. As a result, HPV self-collection could reduce social and health inequities in accessing cervical cancer screening services in low-resource areas with distant health facilities and limited transportation [20]. In addition, studies have shown that self-collected HPV samples are accurate [17, 21, 22], cost-effective [10], feasible [20, 23], acceptable [20, 23], and convenient for women in SSA [24]. However, HPV self-collection is not used in most low- and middle-income countries (LMICs), underlining the need for implementation of scientific research.

While emerging evidence supports the effectiveness, feasibility, and acceptability of HPV self-collection among women in SSA [25, 26], less is known about how these findings can be translated into routine practice in real-world settings. HPV self-collection may be particularly useful in reaching women living in rural settings where there may be limited infrastructure for traditional cervical cancer screening [27]. Evaluating the external and internal validity of HPV self-collection interventions is important for characterizing the generalizability and real-world impacts of HPV self-collection among women in SSA [28]. The Reach, Effectiveness, Adoption, Implementation, and Maintenance (RE-AIM) framework provides a guide for evaluating the real-world impact of public health interventions through a balanced assessment of external and internal validity dimensions that are important in the translation of research to practice [29, 30]. The framework provides a comprehensive guide for disseminating and implementating effective interventions into practice [31, 32]. Specifically, the RE-AIM framework assesses the following: (1) how an intervention reaches the target population and the extent to which the intervention participants are representative of the non-participants; (2) how an intervention achieved the projected objectives, with optimal quality of life; (3) how an intervention was broadly adopted and the extent to which delivery setting and the delivery staff were representative of non-deliverers; (4) how responsible organizations and staff implemented an intervention at a reasonable cost; and (5) an intervention’s ability to be sustained, with long-lasting individual effects [31–34]. RE-AIM has been used in other systematic reviews evaluating the public health impact of HIV [35] and HIV/NCD integration [36] interventions in SSA and has demonstrated utility in bridging the research to practice gap for health interventions [37].

However, a comprehensive review of the internal and external validity of self-collection for HPV testing interventions among women in SSA is currently lacking. This gap in the literature limits the ability to guide the dissemination and implementation of self-collection for HPV testing interventions into practice. Thus, the purpose of this review is to (1) evaluate the extent to which randomized controlled trials aimed at evaluating self-collection for HPB testing in SSA have reported on dimensions on internal and external validity dimensions using the RE-AIM framework and (2) offer guidance on the design and reporting of future self-collection for HPV testing interventions to improve women’s health in the region.

## Methods

We conducted this systematic review following the Preferred Reporting Items for Systematic Review and Meta-Analyses (PRISMA) checklist [38]. The PRISMA checklist is provided in Additional file 1. This systematic review is registered with the PROSPERO international prospective register of systematic reviews (CRD42020214351) at the Centre for Reviews and Dissemination, University of York, UK.

### Search strategy

We searched four electronic bibliographic databases (PubMed, Global Health, Web of Science, the Cumulative Index to Nursing and Allied Health Literature (CINAHL), and EMBASE) for articles published through August 9, 2020, and updated on November 20, 2020. We searched for articles including all three key concepts: (1) HPV self-collection, (2) pragmatic randomized controlled trials focused on intervention implementation, and (3) studies conducted in Sub-Saharan Africa guided by the work by Yeh and colleagues [39]. Keywords and Medical Subject Headings (MeSH) were applied to capture all key concepts, and search terms were modified for each database. See Additional file 2 for the detailed search strategy used for PubMed which was modified and used in other databases. Search terms were limited to English language publications. Also, published systematic reviews [25, 39] focused on self-collection for HPV testing, as well as reference lists from the included articles, were searched to augment the database literature search.

### Study selection

All citations from the initial search were imported into a reference manager, where duplicates were deleted, and titles and abstracts were screened independently by two reviewers (UN, CO-U). The full text of relevant articles was further screened by two independent reviewers (UN, CO-U) using the review inclusion/exclusion criteria. All disagreements regarding article relevance and eligibility were discussed until consensus was reached.

### Eligibility criteria

Studies were selected according to the following inclusion criteria: (a) *Participants:* Women; (b) Intervention: HPV self-collection; Comparators: Comparison of alternative interventions that do not include self-collection for HPV testing (e.g., cervical screening by cytology, Visual Inspection with Acetic Acid (VIA) testing services, clinician-collected primary HPV testing); (c) Outcomes: Uptake of self-collection for HPV testing, acceptability of HPV, frequency of cervical cancer screening, linkage to treatment following positive self-test diagnosis; (d) Study designs: Pragmatic Randomised controlled trials (RCTs) - pragmatic RCTs seek to maximize external validity by providing information on the relative merits or real-world clinical alternatives in routine care [40]; and (e) Location: studies conducted in Sub-Saharan Africa. Studies were excluded from the study if the main focus was cervical cancer screening and not HPV self-collection. We also excluded scoping reviews, systematic reviews, commentaries, and opinion pieces.

### Data extraction and synthesis

Two authors (UN, CO-U) independently piloted a structured extraction from three studies, one other author (TS) critically reviewed, suggested improvements, and approved the final version of the data extraction form used for the review. Data extraction was performed independently by two authors (UN and CO-U), and any inconsistencies were discussed to reach a consensus. The following information was extracted for each study: first author, year of publication, country of study, HPV detection method, sample collection device, population description, participants’ age range, study design, and sample size. Data from the articles included in this review were analyzed using narrative synthesis [41].

For RE-AIM evaluation, we used a 23-item data collection tool adapted from RE-AIM.org and has been used in several previous systematic reviews that reported the RE-AIM dimensions [42]. See Additional file 3 for the definition of the RE-AIM dimensions. Binary coding was used to report whether individual indicators were reported (1) or not reported (0) within each of the five RE-AIM dimensions. Frequencies, proportions, and means were calculated for each of the indicators. The average proportion of indicators reported within each RE-AIM dimension was calculated by summing the number of indicators reported for a given dimension divided by the total number of possible indicators within the dimension. Also, the proportions of each of the 23-item indicators were derived by summing across all studies and dividing by the total number of interventions (*n*=5).

### Risk of bias assessment

We assessed the risk of bias of the included interventions using the Cochrane Collaboration risk of bias tool [43, 44]. The tool consists of the evaluation of six domains: selection bias, performance bias, detection bias, attrition bias, reporting bias, and other biases [43, 44]. Two authors (UN and CO-U) independently rated the risk of bias for the six domains [43, 44] as low, high, or unclear risk. Differences in the risk of bias ratings were resolved through consensus by discussion. The Cochrane Collaboration risk of bias assessment tool was only used to evaluate the internal validity of the interventions included in the review; no study was excluded from the review based on the risk-of-bias score.

## Results

### Search strategy

**Fig. 1:**
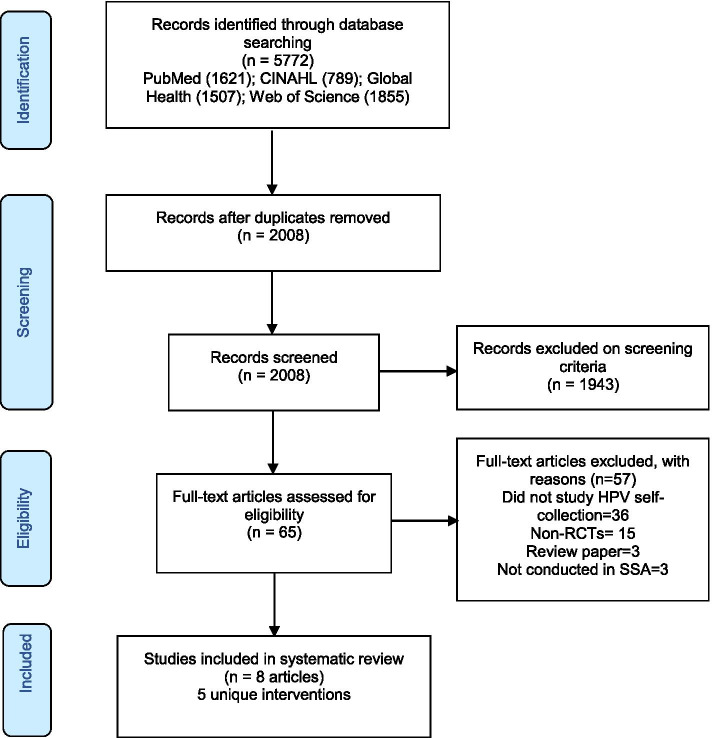
Flow diagram of the search strategy

The original search yielded 2008 potentially eligible articles after duplicates of articles were removed. Of those, 1943 articles were excluded during the title and abstract screening, yielding 65 articles for full-text review. An additional 57 articles were excluded after review of the full text for the following reasons: not focused on self-collection for HPV testing, being non-randomized studies, not in sub-Saharan Africa, and for being review papers. A total of 8 eligible papers, covering 5 unique interventions were finally included in this systematic review. Details on the search strategy are provided in Fig. 1.

### Characteristics of included studies

Table 1 summarizes the characteristics of the intervention studies. The majority of the intervention studies were published within the last 6 years, with the earliest published in 2014 and the latest in 2019. All five interventions included in the systematic review were randomized controlled trials. Overall, study sample sizes ranged from 301 in the intervention study by Sossauer et al. [45] to 4944 in the intervention study by Huchko et al. [46] (Median: 500; IQR 350.5–3121.5). The five interventions were conducted in five distinct countries: Cameroon [45], Ethiopia [47], Kenya [46], Nigeria [48], and Uganda [49]. Two of the interventions recruited women ages 25–65 years [45, 50], another two, recruited women ages 30–65 years [48, 49] and one intervention had a lower limit for recruitment, targeting women ages 30–49 years [47]. Only three of the intervention indicated the type of HPV self-collection kit used: Evalyn Brush (Rovers) in the Gizaw et al., study in Ethiopia [47], Dacron swab used in the study by Moses et al. [49] in Uganda, and careHPV in the study by Huchko et al. [46] in Kenya.

**Table 1:** Summary of intervention characteristics

Author, year	Study location	Population characteristics	Study design	Sample size	Intervention description	Outcomes measures	Results
Gizaw et al., 2019 [47]	Ethiopia	Women Age: 30–49 years	CRCT	1299 (HPV Self-sampling arm:835; VIA arm:464)	Intervention: One arm of the intervention involved self-collection-based HPV DNA testing. Women were offered an Evalyn Brush (Rovers) to collect a swab under active supervision by a trained health professional. Women collected samples in a private area in the health post. Samples were immediately placed in a plastic bag provided by the Evalyn Brush Company after giving a unique ID to the study participants. Control: The other arm of the study completed VIA at a hospital. A trained and certified the nurse was responsible for performing the screening. All women who tested VIA positive were rescreened by a gynecologist for quality assurance.	Uptake of HPV testing	Of those women who attended the VIA and HPV arms, 40%, and 65.4% adhered to all procedures expected after the screening, respectively. Out of women positive for high-risk HPV, 122 (85%) attended VIA as a follow-up test. The trial demonstrated significantly higher levels of population-based uptake and adherence for self-collection HPV testing.
Huchko et al., 2017 [46]; Oketch et al., 2019 [80]; Page et al., 2019 [81]	Kenya	Women Age: 25–65 years	CRCT	4944	Intervention: Cervical cancer screening was carried out using HPV testing of self-collected specimens through community health campaigns Device-*careHPV* Control: HPV testing at health facilities	Uptake of HPV testing	Screening uptake was greater in communities assigned to community health campaigns compared to those assigned to receive screening through health facilities (60.0% vs 37.0%, *P*<0.001).
Modibbo et al., 2017 [48]	Nigeria	Women residing in an urban area Age: 30–65 years	RCT	400 (Intervention group: 200; Control group: 200)	Intervention: HPV self-sampling kit directly mailed to the home address with a prepaid return envelope (or could drop off the completed kit at designated collection points in the community or the hospital). Cervicovaginal specimen, collected at home, unsupervised. Device: Not reported Control: HPV testing appointment at the hospital clinic.	Uptake of HPV testing services Length of follow-up: 1 month	Most participants in the self-collection arm (93%, 185/200) submitted their samples while only 56% (113/200) of those invited to the hospital for sample collection attended and were screened during the study period (*p* value < 0.001).
Moses et al., 2015 [49]; Mezei et al., 2018 [10]	Uganda	Women residing in an urban area Age: 30–65 years	RCT	500 (Intervention group:250; Control group: 250)	Intervention: HPV self-sampling kit and education offered door-to-door by outreach worker (return to the worker). Cervicovaginal specimen, collected at home, unsupervised. Device: Dacron swab. Control: Screened by a healthcare provider (VIA)	Uptake of HPV testing services. Linkage to clinical assessment or HPV treatment	In the HR-HPV arm, 248 of 250 (*p* < 0.01) women provided samples, while in the VIA arm, 121 of 250 (48.4%) women attended the screening. Among the 73 of 248 HR-HPV-positive women, 45.2% (*N* = 33) attended VIA screening for follow-up, 21.2% (*N* = 7) of whom screened positive; five received treatment, and two were missing clinical follow-up records. Of the 121 women in the VIA arm who attended the screening, 13.2% (*N* = 16) screened positive; seven received cryotherapy, three refused treatment, five were referred to colposcopy; and one woman had suspected cervical cancer and received treatment after confirmatory testing.
Sossauer et al., 2014 [45]	Cameroon	Women Age: 25–65 years	RCT	301 (Intervention group:152; Control group: 149)	Intervention: Standard information (this included explanations about what the tests detects (HPV), the link between HPV and cervical cancer, and how to perform HPV self-sampling) followed by the educational intervention (this consisted of a culturally tailored video about HPV, cervical cancer, Self-HPV, and its relevancy as a screening test). Control: Standard information	Knowledge about HPV and acceptability and confidence in using self-HPV	301 women (149 in the “control group” and 152 in the “intervention group”) completed the full process and were included in the analysis. Participants who received the educational intervention had a significantly higher knowledge about HPV and cervical cancer than the control group (p,0.05), but no significant difference in Self-HPV acceptability and confidence in the method was noticed between the two groups.

### Quality of included studies

More than half of the domains of risk of bias were low or unclear across the studies. Selection bias due to randomization was low across the studies as the consistently used random sequences for randomization (20%), except for one of the studies which conducted the randomization after the study enrollment process [48]. Lack of evidence regarding participants and personnel blinding contributed to potential performance and detection bias. Potential performance bias was high (80%), and 20% of the studies had a low detection bias. Potential attrition bias attributed to incomplete data was low (10%). Also, potential reporting bias and bias from other sources such as confounders were found to be low (0%). Details on the quality assessment of the studies are provided in Additional file 4.

### RE-AIM indicators

Overall, individual intervention reported 11 to 15 (median=12) out of a total of 23 RE-AIM indicators. None of the interventions reviewed addressed all 23 indicators across the 5 RE-AIM dimensions. Overall, the average reporting proportions were highest for reach (80%), followed by adoption (56%), efficacy/effectiveness (52%), implementation (47%), and the least reporting rates were for maintenance (0%). Table 2  summarizes the overall percentage of studies reporting on each of the RE-AIM framework dimensions. See Table 2 for additional details on the RE-AIM indicator reporting and Table 3 provides details on the proportion of RE-AIM indicators for the included interventions.

**Table 2:** The reporting on RE-AIM indicators across the studies

RE-AIM dimensions and components	Gizaw et al., 2019 [47]	Huchko et al., 2017 [46]; Oketch et al., 2019 [80]; Page et al., 2019 [81]	Modibbo et al., 2017 [48]	Moses et al., 2015 [49]; Mezei et al., 2018 [10]	Sossauer et al., 2014 [45]
**Reach**					
Method to identify target population	Reported	Reported	Reported	Reported	Reported
Inclusion criteria	Reported	Reported	Reported	Reported	Reported
Exclusion criteria	Reported	Reported	Reported	Reported	Reported
Sample size	Reported	Reported	Reported	Reported	Reported
Participation rate	Not reported	Reported	Reported	Not reported	Not reported
Characteristics of participants	Reported	Reported	Reported	Reported	Reported
Characteristics of non-participants	Reported	Not reported	Not reported	Not reported	Not reported
**Efficacy/effectiveness**					
Measures/results for at least one follow-up	Reported	Reported	Reported	Reported	Reported
Intent to treat utilized	Reported	Not reported	Reported	Reported	Not reported
Quality-of-life (psychosocial) measures	Not reported	Not reported	Not reported	Not reported	Not reported
Baseline activity measured	Not reported	Not reported	Reported	Not reported	Reported
Percent attrition	Not reported	Not reported	Reported	Reported	Reported
**Adoption**					
Description of intervention location	Reported	Reported	Reported	Reported	Reported
Description of staff who delivered intervention	Reported	Reported	Reported	Reported	Reported
Method to identify target delivery agent	Not reported	Reported	Not reported	Not reported	Not reported
Level of expertise of delivery agent	Reported	Reported	Reported	Not reported	Not reported
Adoption rate	Not reported	Not reported	Not reported	Not reported	Not reported
**Implementation**					
Intervention duration and frequency	Not reported	Reported	Reported	Not reported	Reported
Extent protocol delivered as intended	Reported	Not reported	Not reported	Not reported	Not reported
Measures of cost of implementation	Not reported	Not reported	Reported	Reported	Not reported
**Maintenance (3)**					
Assessed outcomes ≥6 months post-intervention	Not reported	Not reported	Not reported	Not reported	Not reported
Current status of program	Not reported	Not reported	Not reported	Not reported	Not reported
Cost of maintenance	Not reported	Not reported	Not reported	Not reported	Not reported

**Table 3:** The proportion of interventions reporting on RE-AIM indicators

RE-AIM dimensions and components	Frequency	Proportion
**Reach**		
Method to identify target population	5	100%
Inclusion criteria	5	100%
Exclusion criteria	5	100%
Sample size	5	100%
Participation rate	2	40%
Characteristics of participants	5	100%
Characteristics of non-participants	1	20%
*Average of overall reach dimensions*	28	80%
**Efficacy/effectiveness**		
Measures/results for at least one follow-up	5	100%
Intent to treat utilized	3	60%
Quality-of-life (psychosocial) measures	0	0%
Baseline activity measured	2	40%
Percent attrition	3	60%
*Average of overall efficacy/effectiveness dimensions*	13	52%
**Adoption**		
Description of intervention location	5	100%
Description of staff who delivered the intervention	5	100%
Method to identify target delivery agent	1	20%
Level of expertise of a delivery agent	3	60%
Adoption rate	0	0%
*Average of overall adoption dimensions*	14	56%
**Implementation**		
Intervention duration and frequency	4	80%
Extent protocol delivered as intended	1	20%
Measures of cost of implementation	2	40%
*Average implementation dimensions*	7	47%
**Maintenance**		
Assessed outcomes ≥6 months post-intervention	0	0%
Status of program	0	0%
Cost of maintenance	0	0%
*Average of overall maintenance dimensions*	0	0%

### Reach

The average proportion reporting across indicators within the reach dimension was 80%. Within the reach dimension, the method to identify the target population 5 (100%), inclusion criteria 5 (100%), sample size 5 (100%), and participants’ characteristics 5 (100%) were reported in all the interventions included in this review. Participants for the intervention studies were identified using a variety of strategies. Two studies identified their target population using demographic and surveillance data. Specifically, the study in Ethiopia identified their target population using the Butajira Health and Demographic Surveillance [47] and the study in Kenya utilized a combination of prospective demographic data, census data, health facility information, and mapping [46]. Community announcements through word-of-mouth [45, 48] and advertisements using posters [45] were also utilized to identify the target population for the intervention study. One study engaged outreach workers to recruit potential participants from their homes and places of work [49]. From a geographical perspective, four of the interventions were conducted in urban areas [45, 47–49] and only one study site was in a rural setting [46].

The inclusion criteria reported in all 5(100%) interventions were mainly focused on the individual being a female, residing in the study region, being within the age requirement for the study, as well as the willingness and ability to provide consent. Exclusion criteria were also reported across the 5 (100%) interventions. Individuals were excluded from the studies if they were pregnant, menstruating, had a previous hysterectomy or cervical surgery, planned to relocate within 6 months, and refused to give consent before the study. The interventions by Modibbo et al. [48] and Huchko et al. [50] also excluded participants who were HIV positive or participating in an HIV testing trial, respectively.

The sample size, defined as the number of participants who participated in the intervention studies, ranged from 301 to 1299. Characteristics of participants commonly reported included age, education level, marital status, religion, and employment status. Two of the interventions recruited women ages 25–65 years [45, 50], another two, recruited women ages 30–65 years [48, 49] and one intervention had a lower limit for recruitment, targeting women ages 30–49 years [47].

The participation rate, determined by the number of participants recruited who participated in the intervention, was reported in two (40%) of the studies. The characteristics of non-participants were also less reported, such that only 1 (20%) of the intervention reported on this reach indicator.

### Effectiveness

On average, the reporting of efficacy/effectiveness indicators was 52% across the five interventions. Within this dimension, the measure or result for at least one follow-up 5 (100%) was the most frequently reported indicator, followed by reporting intent-to-treat 3 (60%) and percent attrition 3 (60%). In terms of measures, the uptake of cervical cancer screening was reported as the primary outcome across the five interventions. All the studies reported higher uptake and adherence for self-collection HPV testing at the end of the study period. In the Sossauer et al. [45] study where the intervention group also received a culturally tailored education on HPV, cervical cancer, and self-collection for HPV testing and the control group received standard information provided at the health centers’, participants in the intervention group had a significantly higher knowledge about HPV and cervical cancer than those in the control group (*p*<0.05. However, there was no significant difference in the acceptability of HPV self-collection and participants’ confidence in completing HPV self-collection between the intervention and control group.

The baseline activity of study participants was reported in 2 (40%) of the studies. The baseline characteristics reported included participants’ indication of previous screening for cervical cancer, sexual behavior including the number of lifetime sexual partners, gynecological history such as the history of the abnormal cervix, and basic socio-demographic characteristics such as age, educational level, and marital status [45, 48]. Intent-to-treat analysis was utilized in 2 (40%) of the interventions to assess intervention uptake at follow-up [47–49]. Three (60%) interventions reported on percent attrition which ranged from 0 [45] to 25% [48]. Attrition resulted from women not completing cervical cancer screening or dropping of the self-collected specimen during the intervention duration. None 0 (0%) of the interventions reported on having measuring the quality of life among study participants.

### Adoption

The average reporting proportion of adoption indicators across studies was 56%. Adoption was assessed at the setting and individual level, including the number, proportion, and description of settings and personnel who participated in delivering the intervention.

With regard to adoption indicators at the setting level. All the interventions were restricted to a specific region, and all 5 (100%) interventions described the location where the intervention was implemented. None of the studies provided information on setting-level inclusion and exclusion criteria. General descriptions of the location, such as the name and population, were provided for each location. The interventions were delivered in community centers and health facilities.

In respect to adoption indicators related to intervention staff, the 5 (100%) interventions provided details on the delivery staff who implemented the intervention. Specifically, trained professional staff such as health providers and local outreach staff assisted with the delivery of various interventions. This consisted of participants’ recruitment, intervention allocation, and health facility-based cervical cancer screening. Among these 5 interventions, only 1 intervention provided detailed information on how the staff for the intervention was identified. The adoption rate at the staff or delivery level and setting level was not reported in any of the studies. None of the articles addressed all criteria for adoption.

### Implementation

The average reporting of implementation indicators across the intervention was 47%. Implementation was assessed by the extent to which studies reported on intervention duration, frequency, fidelity, and cost of implementation. Four (80%) of the interventions reported on the intervention format, which included intervention duration and frequency [45–47]. Among these interventions, three of them specifically included an educational component to educate participants on HPV, cervical cancer, self-collection for HPV testing, as well as a demonstration on how to use the self-collection for HPV testing kits. The intervention by Sossauer and colleagues [45] in Cameroon included a video to provide a visual demonstration of how self-collection for HPV testing works with an opportunity for discussion with participants after the informational. Another unique delivery component among the intervention was seen in the intervention implemented by Hucko et al. [46] in Kenya, where participants who had mobile phones could receive their results from the self-collection for HPV testing through text messages.

Intervention fidelity, or the extent to which the protocol was delivered as intended, was reported by one (20%) intervention. Only Gizaw et al. [47] specifically reported that 40% of participants adhered to all study protocols. The study protocol included participation in the community sensitization program, completing the study questionnaire, completing HPV testing [VIA testing for the comparison arm and HPV self-collection for the intervention arm], and collecting HPV test results [47].

Intervention cost was reported in 2 (40%) of the interventions [48, 49]. In the ASPIRE intervention in Uganda [10, 49], self-collection for HPV testing was found to be the most effective and cost-effective screening strategy compared to VIA. Specifically, self-collection for HPV testing was reported to reduce the lifetime absolute risk of cervical cancer from 4.2 to 3.5%, with incremental cost-effectiveness ratios (ICERs) of US$130 per dollar per year of life saved (YLS), US$240 per YLS, and US$470 per YLS when performed one, three and five times per lifetime, respectively [10, 49].

### Maintenance

None of the interventions reported on any of the three maintenance indicators (“assessment of outcomes ≥6 months post-intervention,” “the current status of the program,” and “cost of maintenance”).

## Discussion

The primary aim of our review was to systematically assess the implementation of HPV self-collection interventions in SSA. Our analysis highlights the lack of implementation research on HPV self-collection in the region. Of the five self-collection for HPV testing RCTs identified in our review, on average, 11 (47%) of the 23 RE-AIM indicators were reported. To date, the research literature has been directed towards the evaluation of the effectiveness of self-collection for HPV testing. However, major knowledge gaps exist in our understanding of the process of implementation and maintenance of self-collection for HPV testing interventions among women in SSA. Consistent and detailed reporting of the intervention delivery is crucial to enhance the impact of these interventions, generalizability of findings, and potential for scale-up.

We found that many HPV self-collection studies reported internal validity measures [45, 47–50]. Consistent with other reviews using the RE-AIM framework [35, 51], the majority of the studies in our review reported on the methods used to identify the target populations, sample size, and characteristics of participants. However, the participation rate and characteristics of non-participants, components that reflect external validity, were rarely reported in studies. This limits the generalizability of the data beyond the type of participants in the study. These indicators are vital for understanding the contextual factors that may influence women’s participation in cervical cancer screening [52]. Additionally, limited reporting on characteristics of non-participants limits the ability to identify populations that are not engaged in or being reached by these interventions. In an effort to address broad access to interventions and include subgroups of the target population that are most in need of such interventions, researchers need to improve on the reporting of the characteristics of non-participants in an intervention study [35]. One way to do so is to utilize a participatory approach involving end-users of these interventions in designing and implementating interventions adapted to their contexts and needs [53, 54]. Given the momentum towards decentralizing STI services to non-clinical settings [55], participatory strategies such as crowdsourcing which invites end-users to brainstorm ideas and solutions to public health issues and then promotes these solutions to end-users [56, 57] may generate knowledge on factors that enhance participation and/or non-participation in self-collection for HPV testing interventions.

Additionally, and similar to the findings from other reviews, effectiveness based upon changes in the primary outcome (i.e., HPV testing uptake) were reported across all studies [58, 59]. Findings from our review highlight the impact of self-collection for HPV testing interventions on cervical cancer screening, with all five studies reporting a significant improvement in some measure of uptake of cervical cancer screening. Measures of effectiveness were the most commonly reported component of the efficacy/effectiveness dimensions, while the quality of life (psychosocial measures) and baseline activity were the least reported. Particularly, the quality of life measures provides a metric to compare across interventions with different behavioral targets and provides a better sense of the impact that the intervention has on the participants’ perceptions of health [28, 60]. Given that HPV-self-collection is relatively a novel area of research in SSA, the focus may have been on determining the impact on screening uptake with scarce reporting of implementation indicators [61]. Moreover, the positive effects reported in these studies may have been overestimated as not all the included studies considered the extent of and reasons for attrition. Yet, why some women participate or choose not to participate in these interventions has important implications for reducing the burden of cervical cancer globally [62, 63]. By omitting such details, an opportunity is lost to further understand barriers or challenges that influence the continued participation of women in cervical screening programs in low-resource settings [64, 65].

Regarding adoption, most of the intervention studies described the intervention location and staff who delivered the intervention. However, only one intervention described the methods used to identify intervention delivery agents [50] and no study provided the adoption rate of the intervention at both individual and setting levels. Yet, the adoption rate matters. Limited information on characteristics of the individual delivery agents and settings within which these interventions take place has implications for the translation of self-collection for HPV testing into real-world settings within the region [35]. It limits the understanding of the characteristics of the settings that work well or may not work well to optimize the implementation of self-collection for HPV testing [35]. Additionally, it limits the identification of factors that may influence translation into practice both within clinical and non-clinical settings and among individual patients.

Although we found limited reporting on the implementation dimension of the RE-AIM framework. Studies included in our review commonly reported the intervention duration and frequency, a consistent finding of other intervention studies [35, 51, 66], which enhances the replication of intervention delivery within real-world practice [51]. However, fidelity, or the extent to which the protocol was delivered as intended and the cost of implementation, was the least reported components of the implementation dimension. Failing to address fidelity with self-collection for HPV testing interventions may have an adverse impact on the effectiveness of these interventions and ultimately perpetuate the burden of cervical cancer [67]. Additionally, policymakers’ appraisal of the cost of self-collection for HPV testing can inform decisions about funding and resource allocation, which makes information on intervention cost-effectiveness critical for making decisions on the scale-up of self-collection for HPV testing interventions in SSA [68, 69].

Finally, and consistent with prior studies [3, 42, 51, 70], none of the intervention studies included in our review reported the maintenance of intervention effects whether at individual or setting levels. Similar to adoption, maintenance whether at the individual or setting level has implications for reducing health disparities related to the cervical cancer burden among women in SSA [3]. Measuring maintenance allows researchers and policymakers to determine whether an effective intervention should be disseminated or scaled up widely [54]. Data on maintenance also allows for an understanding of the contextual determinants or processes necessary for sustaining interventions [71]. The WHO recommends regular HPV-based testing as one of the screening methods for cervical cancer at 3- or 5-year intervals depending on other criteria [72]. As such, sustaining HPV-self collection interventions will be key to significantly reducing the burden of cervical cancer in the region.

There are a few implications and recommendations based on the findings of this review. Future intervention research studies should consider reporting on intervention implementation to enhance the application and translation to real-world contexts. This review highlighted missing opportunities in reporting on intervention adoption and sustainability and key information for uptake of study findings in practice and policy. The use of implementation science frameworks as a guide for intervention development and implementation can enhance the translation of research findings into practice. This is paramount for the effective adoption and scale-up of HPV testing within routine cervical cancer screening, specifically the self-collection approach, shown to have an overall significant higher uptake based on findings from intervention studies included in this review.

### Limitations and strengths

There are limitations to this review worth mentioning. First, the conclusions of our review are based on the extent to which the included studies reported on the RE-AIM dimensions. Therefore, some studies may have collected this information, but not reported it in the main research manuscript. Additionally, the focus of our review was reporting of RE-AIM dimensions which may be different from the main purposes of the RCTs included in the review to assess the effectiveness of HPV-self collection on uptake of cervical cancer screening, which focused more on the internal validity of the studies. Second, we focused on reporting the indicators across RE-AIM dimensions, which are different from efficacy-based study quality evaluation that assess the adequacy of study design, sample size, participants’ randomization, and use of validated metrics and statistical methods [51]. Therefore, studies that would typically score high on these efficacy-based study quality evaluations may have scored low when evaluated with the RE-AIM evaluation framework and vice versa [51]. However, it is important to note that our review focused on assessing self-collection for HPV testing interventions using an implementation science lens. Finally, we limited our search strategy to published studies and those available in English, excluding other studies. However, there is empirical evidence that removing non-English studies does not bias systematic review findings [73]. Additionally, we conducted an exhaustive search strategy using well-defined inclusion and exclusion criteria based on the PRISMA guidelines and data extraction tool for the RE-AIM framework [35].

## Conclusion

This systematic review makes a unique contribution to the literature on whether it is time to RE-AIM the reach, effectiveness, adoption, implementation, and maintenance of self-collection for HPV testing interventions among women in SSA. Our findings underscore the need for researchers to tailor their research designs to maximize the reporting of external validity factors. Innovative tools such as participatory learning approaches or open challenges [74–77] to expand knowledge of external validity indicators are also warranted to effectively enhance the reach, adoption implementation, and long-term maintenance of self-collection for HPV testing among women in SSA. The poor reporting on these components within all dimensions of the RE-AIM framework may contribute to the limited widespread dissemination of effective self-collection for HPV testing interventions in the region. As a result, efforts are needed to design self-collection for HPV testing strategies that are participatory, with end-users themselves guiding ways to expand the reach, adoption, and implementation of these interventions [53, 78, 79]. Such studies produce sustained and equitable outcomes that are adapted to the local contexts and needs of participants and community settings where the burden of cervical cancer remains high.

## Supplementary Information

**Additional file 1.** Preferred Reporting Items for Systematic Review and Meta-Analyses (PRISMA) checklist.

**Additional file 2.** Search strategy for PubMed which was modified and used in other databases.

**Additional file 3.** Definition of the dimension on the RE-AIM framework [1, 2].

**Additional file 4.** Quality of Included Studies based on Cochran Collaboration risk of bias tool [1, 2].

## Data Availability

Not applicable.

## Abbreviations

CO-J: Chisom Obi-Jeff
CO-U: Chisom Obiezu-Umeh
DO: David Oladele
HPV: Human papillomavirus
ICERS: Incremental cost-effectiveness ratios
II: Ifeoma Idigbe
JI: Juliet Iwelunmor
JT: Joseph Tucker
LMICs: Low- and middle-income countries
Mesh: Medical Subject Headings
NCD: Noncommunicable diseases
OE: Oliver Ezechi
PRISMA: Preferred Reporting Items for Systematic Reviews and Meta-Analyses
RCTs: Randomized control trials
RE-AIM: Reach, Effectiveness, Adoption, Implementation, and Maintenance
SSA: Sub-Saharan Africa
TG: Titilola Gbaja-biamila
TS: Thembekile Shato
UN: Ucheoma Nwaozuru
VIA: Visual inspection with acetic acid
YLS: Year of life saved
