# Supplementary material for: A systematic review of randomized control trials of HPV self-collection studies among women in sub-Saharan Africa using the RE-AIM framework
Source: Implement Sci Commun. 2021 Dec 15;2:138. doi: 10.1186/s43058-021-00243-5 (PMC8672475; doi:10.1186/s43058-021-00243-5)
Supplement: Supplementary file 2 — Additional file 2. Search strategy for PubMed which was modified and used in other databases. [file 43058_2021_243_MOESM2_ESM.docx]

Additional File 2: Search strategy for PubMed which was modified and used in other databases

| **Category** | **Search Terms Combined with AND** |
| --- | --- |
| Disease-specific terms (HPV and cervical cancer) | ("human papillomavirus” [MeSH] OR HPV [Mesh] OR Cervical OR “human papilloma virus” OR “cervical cancer” OR cervical OR “cervical neoplasia” OR “cervix cancer”) |
| Self collection terms | ( test OR testing OR "self-test" OR "self-testing" OR "home-based test" OR "home-based testing"[tiab] OR "home test" OR "home testing" OR "self-administer" OR "selfsampling" OR "self-collecting" OR "self-collected" OR "self-collection" OR "selfversus provider-collected" OR "self- and provider-collected" OR "self- versus physiciancollected" OR "self- and physician-collected”) |
| Region/countries | ( “Africa” [MeSH] OR “Sub-Saharan Africa” OR Africa OR Cameron OR “the central African Republic” OR Chad OR Congo OR “the Democratic Republic of the Congo” OR “Equatorial Guinea” OR Gabon OR “Eastern Africa” OR Burundi OR Djibouti OR Eritrea OR Ethiopia OR Kenya OR Rwanda OR Somalia OR Sudan OR Tanzania OR Uganda OR Southern Africa OR Angola OR Botswana OR Lesotho OR Malawi OR Mozambique OR Namibia OR South Africa OR Swaziland OR Zambia OR Zimbabwe OR Western Africa OR Benin OR Burkina Faso OR “Cape Verde” OR “Cote d'Ivoire” OR “Ivory Coast” OR Gambia OR Ghana OR Guinea OR “Guinea-Bissau” OR Liberia OR Mauritania OR Niger OR Nigeria OR Senegal OR Sierra Leone OR Togo) |

Note: MeSH = medical subject heading
