# Supplementary material for: A systematic review of randomized control trials of HPV self-collection studies among women in sub-Saharan Africa using the RE-AIM framework
Source: Implement Sci Commun. 2021 Dec 15;2:138. doi: 10.1186/s43058-021-00243-5 (PMC8672475; doi:10.1186/s43058-021-00243-5)
Supplement: Supplementary file 3 — Additional file 3. Definition of the dimension on the RE-AIM framework [1, 2]. [file 43058_2021_243_MOESM3_ESM.docx]

Additional File 3: Definition of the dimension on the RE-AIM framework [1, 2]

| **Implementation outcomes** | **Definition** | **Level** | **Indicators for this Review** |
| --- | --- | --- | --- |
| Reach | The absolute number, proportion, and representatives of individuals who are willing to participate in a given intervention | Individual | - Method to identify target population - Inclusion criteria - Exclusion criteria - Sample size - Participation rate - Characteristics of participants - Characteristics of non-participants |
| Effectiveness | The impact of an intervention on important outcomes | Individual | - Measures/results for at least one follow-up - Intent to treat utilized - Quality-of-life (psychosocial)   measures   - Baseline activity measured - Percent attrition |
| Adoption | The absolute number, proportion, and representativeness of settings, and people who deliver the program who are willing to initiate the program | Setting | - Description of intervention location - Description of staff who delivered intervention - Method to identify target delivery agent - Level of expertise of delivery agent - Adoption rate |
| Implementation | The intervention agents’ fidelity to the various elements of an intervention protocol including consistency of delivery, the time required, and implementation strategy. | Individual & Setting | - Intervention duration and frequency - Extent protocol delivered as intended - Measures of cost of implementation |
| Maintenance | The extent to which behavior is sustained or program is institutionalized 6 months or more after the intervention. Includes reasons for maintenance, discontinuation, or adaptation. | Individual & Setting | - Assessed outcomes ≥6 months post-intervention - Current status of program - Cost of maintenance |

References

1. Glasgow RE, Vogt TM, Boles SM: **Evaluating the public health impact of health promotion interventions: the RE-AIM framework**. *American journal of public health* 1999, **89**(9):1322-1327.

2. Glasgow RE, Harden SM, Gaglio B, Rabin BA, Smith ML, Porter GC, Ory MG, Estabrooks PA: **RE-AIM Planning and Evaluation Framework: Adapting to New Science and Practice with a Twenty-Year Review**. *Frontiers in public health* 2019, **7**:64.
