# Supplementary material for: A systematic review of randomized control trials of HPV self-collection studies among women in sub-Saharan Africa using the RE-AIM framework
Source: Implement Sci Commun. 2021 Dec 15;2:138. doi: 10.1186/s43058-021-00243-5 (PMC8672475; doi:10.1186/s43058-021-00243-5)
Supplement: Supplementary file 4 — Additional file 4. Quality of Included Studies based on Cochran Collaboration risk of bias tool [1, 2]. [file 43058_2021_243_MOESM4_ESM.docx]

Additional File 4: Quality of Included Studies based on Cochran Collaboration risk of bias tool [1, 2]

|  | **Selection bias (Random sequence generation)** | **Selection bias (Allocation concealment)** | **Performance bias (Blinding of participants and personnel)** | **Detection bias  (Blinding of outcome assessment)** | **Attrition bias  (Incomplete outcome data)** | **Reporting bias  (Selective reporting)** | **Other sources of bias** | **Level of Risk** |
| --- | --- | --- | --- | --- | --- | --- | --- | --- |
| Gizaw et al., 2019 [3] | Low | Unclear | High | Unclear | Low | Low | Low | Low Risk |
| Huchko et al., 2017 [4];  Oketch et al., 2019 [5];  Page et al., 2019 [6] | Low | Low | High | Unclear | Low | Low | Low | Low Risk |
| Modibbo et al., 2017 [7] | High | Unclear | Low | Low | Low | Low | Low | Low Risk |
| Moses et al., 2015 [8]; Mezei et al., 2018 [9] | Low | Low | High | Low | Unclear | Low | Low | Low Risk |
| Sossauer et al., 2014 [10] | Low | Unclear | High | Low | Low | Low | Low | Low Risk |

References

1. Higgins JP, Green S: **Cochrane handbook for systematic reviews of interventions**, vol. 4: John Wiley & Sons; 2011.

2. Higgins JP, Altman DG, Gøtzsche PC, Jüni P, Moher D, Oxman AD, Savović J, Schulz KF, Weeks L, Sterne JA: **The Cochrane Collaboration’s tool for assessing risk of bias in randomised trials**. *Bmj* 2011, **343**:d5928.

3. Gizaw M, Teka B, Ruddies F, Abebe T, Kaufmann AM, Worku A, Wienke A, Jemal A, Addissie A, Kantelhardt EJ: **Uptake of cervical cancer screening in Ethiopia by self-sampling HPV DNA compared to visual inspection with acetic acid: a cluster randomized trial**. *Cancer Prevention Research* 2019, **12**(9):609-616.

4. Huchko MJ, Sneden J, Sawaya G, Smith-McCune K, Maloba M, Abdulrahim N, Bukusi EA, Cohen CR: **Accuracy of visual inspection with acetic acid to detect cervical cancer precursors among HIV-infected women in Kenya**. *International journal of cancer* 2015, **136**(2):392-398.

5. Oketch SY, Kwena Z, Choi Y, Adewumi K, Moghadassi M, Bukusi EA, Huchko MJ: **Perspectives of women participating in a cervical cancer screening campaign with community-based HPV self-sampling in rural western Kenya: a qualitative study**. *BMC women's health* 2019, **19**(1):N.PAG-N.PAG.

6. Page CM, Ibrahim S, Park LP, Huchko MJ: **Patient factors affecting successful linkage to treatment in a cervical cancer prevention program in Kenya: A prospective cohort study**. *PloS one* 2019, **14**(9):e0222750.

7. Modibbo F, Iregbu K, Okuma J, Leeman A, Kasius A, de Koning M, Quint W, Adebamowo C: **Randomized trial evaluating self-sampling for HPV DNA based tests for cervical cancer screening in Nigeria**. *Infectious agents and cancer* 2017, **12**(1):11.

8. Moses E, Pedersen HN, Mitchell SM, Sekikubo M, Mwesigwa D, Singer J, Biryabarema C, Byamugisha JK, Money DM, Ogilvie GS: **Uptake of community‐based, self‐collected HPV testing vs. visual inspection with acetic acid for cervical cancer screening in K ampala, U ganda: preliminary results of a randomised controlled trial**. *Tropical Medicine & International Health* 2015, **20**(10):1355-1367.

9. Mezei AK, Pedersen HN, Sy S, Regan C, Mitchell-Foster SM, Byamugisha J, Sekikubo M, Armstrong H, Rawat A, Singer J: **Community-based HPV self-collection versus visual inspection with acetic acid in Uganda: a cost-effectiveness analysis of the ASPIRE trial**. *BMJ open* 2018, **8**(6).

10. Sossauer G, Zbinden M, Tebeu P-M, Fosso GK, Untiet S, Vassilakos P, Petignat P: **Impact of an educational intervention on women's knowledge and acceptability of human papillomavirus self-sampling: a randomized controlled trial in Cameroon**. *PloS one* 2014, **9**(10):e109788.
